# Genome-Wide Identification of Circular RNAs in *Arabidopsis thaliana*

**DOI:** 10.3389/fpls.2017.01678

**Published:** 2017-09-27

**Authors:** Gang Chen, Jiawen Cui, Li Wang, Yingfang Zhu, Zhaogeng Lu, Biao Jin

**Affiliations:** ^1^College of Bioscience and Biotechnology, Yangzhou University, Yangzhou, China; ^2^College of Horticulture and Plant Protection, Yangzhou University, Yangzhou, China; ^3^Department of Horticulture and Landscape Architecture, Purdue University, West Lafayette, IN, United States; ^4^Agricultural College, Yangzhou University, Yangzhou, China; ^5^Key Laboratory of Plant Functional Genomics of the Ministry of Education, Yangzhou, China

**Keywords:** circular RNA, parent gene, *Arabidopsis*, RNA-seq, sequence conservation, microRNA

## Abstract

Circular RNAs (circRNAs) are a family of transcripts with covalently closed circular structures and still largely unknown functions. Large numbers of circRNAs have been found in various biological processes in humans and animals, but fewer circRNAs have been identified in plants. We performed a genome-wide analysis of circRNAs in *Arabidopsis thaliana* via deep sequencing. We constructed 14 strand-specific libraries from 13 samples of plants from four developmental stages, four stress treatments, and five organs and a mixed sample across the lifespan. In total, we identified 5861 circRNAs, including 1275 novel ones, using the strict threshold of at least two unique back-spliced supporting reads. The circRNAs were non-randomly distributed in all chromosomes; most were exonic. Sequence similarity analysis of circRNAs between *A. thaliana* and four other species showed that some circRNAs are conserved in plants. Functional annotation indicated that many parent genes of circRNAs are involved in many fundamental processes including plant development, reproduction, and response to stimulus. In addition, a small proportion of circRNAs was shown to be potential targets of miRNAs, indicating that the circRNAs could interact with miRNAs to regulate gene expression. qRT-PCR analysis revealed that circRNAs displayed diverse expression patterns at different growth stages. Our results provide an important resource for continuing circRNA research in *A. thaliana*, and should enhance our understanding of circRNAs in plants.

## Introduction

There are two types of RNA in eukaryotic cells: messenger RNA (mRNA) and non-coding RNA. Non-coding RNA includes highly abundant and functionally important RNAs, such as transfer RNA (tRNA) and ribosomal RNA (rRNA), as well as microRNA (miRNA), small interfering RNA (siRNA), *trans*-acting siRNA (tasiRNA), small nuclear RNA (snRNA), small nucleolar RNA (snoRNA), and long non-coding RNA (lncRNA). Circular RNAs (circRNAs), which are generally recognized as another group of non-coding RNAs, are characterized by the presence of a covalent bond linking the 3′ and 5′-ends generated by back-splicing (Jeck et al., 2013). CircRNAs arise from back-spliced exons, introns as circular intronic RNAs (ciRNAs), intergenic regions, or both exons and introns as exon–intron circRNAs (EIciRNAs) (Zhang et al., 2013; Li et al., 2015). In past decades, circRNAs have only been occasionally identified due to limitations of molecular techniques and bioinformatic tools (Sanger et al., 1976; Hsu and Coca-Prados, 1979; Grabowski et al., 1981; Kos et al., 1986; Nigro et al., 1991), and were thought to be functionless by-products of transcription. In recent years, with the development of high-throughput sequencing technology and high-efficiency large-scale data analysis, many circRNAs have been identified in archaea, *Caenorhabditis elegans*, mouse, and human (Danan et al., 2012; Salzman et al., 2012; Memczak et al., 2013; Zhang et al., 2013); knowledge of circRNAs has been updated continuously and rapidly.

Recent research has suggested that circRNAs are widely expressed in a complex tissue-, cell-type- or developmental-stage-specific manner, and their sequences and expression patterns are often conserved (Salzman et al., 2012; Jeck et al., 2013; Memczak et al., 2013; Zhang et al., 2013, 2014). The conservation feature of circRNAs exist in various species (Wang et al., 2014; Zhao et al., 2017), and some conserved circRNAs were derived from important gene loci suggesting their potentially important functions (Vicens and Westhof, 2014; Gao et al., 2015). Many circRNA expression levels can be approximately 10-fold compared with their linear isoforms, suggesting that the formation of circRNAs may be finely regulated (Rybak-Wolf et al., 2015; Venø et al., 2015; Dang et al., 2016). Furthermore, circRNAs play important biological roles in transcriptional and post-transcriptional regulatory networks governing gene expression. Functional circRNAs have been shown to act as cytoplasmic miRNA sponges that sequester miRNAs (Hansen et al., 2013; Memczak et al., 2013) and RNA-binding protein sequestering agents (Zhang et al., 2013) as well as nuclear transcriptional regulators (Li et al., 2015). In addition, the production of circRNAs plays an important role in the regulation of alternative splicing (Zhang et al., 2014; Gao et al., 2016). Recent studies have shown that circRNAs serve as protein-coding sequences *in vitro* and *in vivo* (Granados-Riveron and Aquino-Jarquin, 2016; Legnini et al., 2017; Pamudurti et al., 2017; Yang et al., 2017). Studies have also demonstrated the important role of circRNAs as biomarkers of disease, including Alzheimer’s disease (AD) and cancer (Lukiw, 2013; Li et al., 2015).

Although it has been demonstrated that circRNAs play important roles in a range of biological and developmental processes in animals (Memczak et al., 2013; Salzman et al., 2013), the knowledge of circRNAs in plants is limited (Wang et al., 2014; Lu et al., 2015; Ye et al., 2015; Darbani et al., 2016; Sablok et al., 2016; Wang et al., 2017). Wang et al. (2014) reported that circRNA is expressed in a wide range of eukaryotic species, including the model plant species *Arabidopsis thaliana*. Ye et al. (2015) used publicly available RNA sequencing data and anchor aligned the reads to the model plants *Oryza sativa* and *A. thaliana* revealing a large number of circRNAs in *O. sativa* and *A. thaliana*. A study in *O. sativa* by Lu et al. (2015) identified 2,354 circRNAs, among which 1,356 were exonic circRNAs. Recently, a plant circRNA database (PlantcircBase) was created by collecting publicly available and newly identified circRNA sequences from *O. sativa*, *A. thaliana*, *Zea mays*, *Solanum lycopersicum*, and *Hordeum vulgare* (Chu et al., 2017).

Here, to uncover a more comprehensive profile of circRNAs in the plant model species *A. thaliana*, we identified and analyzed circRNAs from 14 ssRNA-seq (strand-specific sequencing of RNA) libraries from RNA samples corresponding to four different growth stages, five organs, and four stress treatments, as well as a mixed RNA sample, in *A. thaliana*. We then used deep sequencing by the Illumina ssRNA-seq approach: our average sequencing depth reached approximately 106× and we acquired high-quality sequence information for circRNAs. In particular, we used the strict threshold of at least two unique back-spliced supporting reads to positively identify circRNAs. In addition, functional annotation analysis and circRNA-originating target miRNA predictions were made to predict the function of circRNA in *A. thaliana*. Our data provide a genome-wide profiling of *A. thaliana* circRNAs and provide an important resource for future circRNA research in *A. thaliana*.

## Materials and Methods

### Plant Materials

Seeds of *A. thaliana* [wild-type Columbia (Col-0)] were exposed to stratification for 2 days at 4°C, and sown in square surface-sterilized plastic pots (7 × 7 × 8 cm) containing sterile medium (1:1 v/v mixture of vermiculite and peat). Pots were arranged in a plastic pallet and placed in a growth chamber (23°C during the day and 18°C at night, with a 16-h photoperiod and 500 μmol m^-2^ s^-1^ of photosynthetically active radiation (PAR); the plants were alternately watered to saturation with distilled water or 1/2 Murashige–Skoog solution (Jin et al., 2011).

Fourteen plant materials for RNA isolation were prepared as follows (**Table 1**). Plants at growth stages 1.04 (4 rosette leaves > 1 mm in length), 1.14 (14 rosette leaves > 1 mm in length), 3.90 (rosette growth complete), 5.10 (first flower buds visible), 6.50 (50% of flowers to be produced have opened), 8.00 (first silique shattered), and 9.70 (senescence complete; ready for seed harvest) (Boyes et al., 2001) were flash-frozen in liquid nitrogen. We used three plants in most of the growth stages, but five plants were sampled in the first stage for isolation of sufficient RNA. For organ sampling, we collected roots, stems, leaves, flowers, and siliques from three plants at stage 6.50. In addition, seedlings at stage 3.90 were exposed to the following stress environments: 300 mM mannitol (drought), 200 mM NaCl (salinity), and 38°C (heat). Plants grown in the same environment (3.90), without the additional stress component, were used as controls. The whole plants were collected 12 h after exposure to stress.

**Table 1:** Summary of *A. thaliana* samples used for ssRNA sequencing.

Sample No.	Description
Arab_mix	Mixed sample from whole plants at seven growth stages: 1.04 (4 rosette leaves > 1 mm in length), 1.14 (14 rosette leaves > 1 mm in length), 3.90 (rosette growth complete), 5.10 (first flower buds visible), 6.50 (50% of flowers to be produced have opened), 8.00 (first silique shattered), and 9.70 (senescence complete; ready for seed harvest).
Arab_B673-T01	Three whole plants at growth stage 1.14
Arab_B673-T02	Three whole plants at growth stage 3.90
Arab_B673-T03	Three whole plants at growth stage 6.50
Arab_B673-T04	Three whole plants at growth stage 8.00
Arab_B673-T05	Roots from three plants at growth stage 6.50
Arab_B673-T06	Stems from three plants at growth stage 6.50
Arab_B673-T07	Leaves from three plants at growth stage 6.50
Arab_B673-T08	Flowers from three plants at growth stage 6.50
Arab_B673-T09	Siliques from three plants at growth stage 6.50
Arab_B673-T10	Aerial part of three plants without any stress treatments at growth stage 3.90
Arab_B673-T11	Aerial part of three plants with drought stress at growth stage 3.90
Arab_B673-T12	Aerial part of three plants with salt stress at growth stage 3.90
Arab_B673-T13	Aerial part of three plants with heat stress at growth stage 3.90

### RNA Extraction and RNase R Treatment

Total RNAs from all samples above were extracted using the MiniBEST Plant RNA Extraction Kit (Takara, Dalian, China), in accordance with the manufacturer’s protocol. We stored the total RNA aliquots at -80°C with 1 unit/μL RNaseOUT (Invitrogen), validated the RNA quality using an Agilent 2100 Bioanalyzer, and quantified the total RNA with Qubit 2.0 Fluorometer (Invitrogen). The qualified total RNAs from the seven growth stages were equally mixed as a pool, while total RNAs from four different growth stages, five organs, and four stress treatments were separately prepared for library construction. Then, the 14 RNA samples were depleted of 18S and 28S rRNA using the Ribo-Zero Magnetic Kit (Epicentre, Madison, WI, United States) and treated with Ribonuclease R (Epicentre, Madison, WI, United States) to remove linear RNA.

### Library Preparation and Illumina Sequencing

The rRNA-depleted RNAs were first fragmented into short sequences in fragmentation buffer at 94°C for 15 min. Second, these fragments were used as templates for first-strand cDNA synthesis primed with random hexamers. The second-strand cDNA was synthesized using buffer, dNTPs, RNase H, and DNA polymerase I, followed by AMPure XP bead purification. Third, the short fragments were further resolved with NEBNext End Repair Reaction Buffer and NEBNext Pre Enzyme Mix for end repair and poly (A) ligation. Subsequently, the fragments were connected with adapters, and then the second strand containing “U” was degraded using uracil-specific excision reagent (USER). Finally, suitable fragments were selected as templates for the PCR amplification after agarose gel electrophoresis. The quantification and qualification of the libraries were further assessed using an Agilent 2100 Bioanalyzer (Agilent Technologies, Palo Alto, CA, United States) and Qubit 2.0 Fluorometer (Invitrogen). These libraries were paired-end (PE) sequenced using the Illumina HiSeq 2500 (read length, PE125, the mixed sample) and Illumina Hiseq X Ten (read length, PE150, 13 independent samples) platforms. All raw sequence data have been deposited in the NCBI Sequence Read Archive (SRA, accession number SRP069764).

### Read Alignment and Analysis

We used the NGS QC Toolkit v2.3.3 software to filter the reads with adapter sequences, reads with an unknown base (N, unknown bases in a read > 5%), and low-quality reads (reads with base quality ≤ 20). Sequence alignments were performed using TopHat2 with BOWTIE2 (v2.0.5) (Langmead and Salzberg, 2012) software for mapping to the reference genome (*Arabidopsis_thaliana* TAIR10.30 ENSEMBL). The circRNAs were predicted using the find_circ program with the same parameters as described previously (Memczak et al., 2013). Chimeric mapped reads were selected for circular (“back-spliced”) junctions if the sequence reads mapped to one chromosome on the same strand, while the two sequence segments mapped to the genomic region with reverse order. CircRNA abundance was predicted on the basis of circular junction read counts. The overlapping region between predicted circRNAs and the gene region of the *A. thaliana* reference genome was identified using Bedtools software (Quinlan and Hall, 2010), and the circRNAs with overlapping regions were annotated. All expression data and other information (coordinates, strand, etc.) of identified *A. thaliana* circRNAs are in GSE77672. To evaluate the conservation of circRNAs, the circRNA sequences of *A. thaliana*, *O. sativa*, *H. vulgare*, *S. lycopersicum*, and *Z. mays* were first downloaded from PlantcircBase^1^. Then, our identified circRNA sequences (Supplementary Table S6) from *A. thaliana* were used for a BLAST search (BlastN in BLAST+, v2.2.27, *E* < 1e^-5^) against the circRNA sequences from *A. thaliana*, *O. sativa*, *H. vulgare*, *S. lycopersicum*, and *Z. mays*.

To evaluate the potential functions of the parent genes of circRNAs, the parent mRNAs were used for BLAST searches and functionally categorized according to gene ontology (GO) annotation by BLAST2GO software with the default parameters^2^ (Conesa and Götz, 2008). To predict miRNA–circRNA interactions, TargetFinder^3^ was used to scan the miRNA-target sites of circRNA. The network of miRNAs–circRNAs was generated using Cytoscape 3.5.1 software.

### PCR Amplification, Sanger Sequencing, and Quantitative Real-Time PCR

Treated RNA solutions (10 μL, without DNA contamination) from seven stages, leaves, and roots were subjected to reverse transcriptase reactions with the PrimeScript RT Reagent Kit (Takara, Dalian, China), in accordance with the manufacturer’s protocol. Genomic DNA of *A. thaliana* was extracted using the Plant DNA Isolation Reagent (Takara, Dalian, China). To validate circRNAs identified in *A. thaliana*, polymerase chain reactions (PCRs) were performed using a set of divergent primers and a set of convergent primers that were used as a control (Supplementary Table S1). The divergent primers were designed using an “out-facing” strategy to guarantee that the amplifications were from a circular template (Shen et al., 2015). For each PCR amplification, 20 ng of cDNA or genomic DNA was used with rTaq DNA polymerase and 10× buffer (Takara, Dalian, China), and 35 cycles of PCR were performed. To confirm the PCR results, the PCR products were dissected from a gel and purified using MiniBEST Agarose Gel DNA Extraction Kit Ver. 4.0 (Takara, Dalian, China). Sanger sequencing was performed by Sangon Biotech Company (Shanghai, China).

A qRT-PCR experiment was performed to test the expression levels of circRNAs. The qRT-PCR reactions contained 1 μL of diluted cDNA, 400 nM of each primer, 10 μL of the 2× TransStart Tip Green qPCR SuperMix, 0.4 μL of the 50× passive reference dye (TransGen Biotech, Beijing, China), and 7.8 μL of ddH_2_O, for a final volume of 20 μL. The following qRT-PCR program was used: denaturation at 94°C for 30 s, followed by 40 cycles of 94°C for 5 s, 55.5°C for 30 s, and 72°C for 10 s. The divergent primers used in the experiment are shown in Supplementary Table S1. Amplification results were analyzed using the comparative Ct method, which uses the formula 2^-ΔΔC_T_^. Each qRT-PCR experiment was carried out in three independent biological replicates and the standard errors of the mean among the replicates were calculated. Statistical analysis was conducted using the SPSS Statistics 18.0 software (IBM Corporation, Armonk, NY, United States). The significance of differences between leaves and roots was analyzed using a *t*-test at the probability level of 0.05.

## Results

### Identification of CircRNAs in *A. thaliana*

RNAs were extracted from 14 samples (13 separate samples and 1 mixed sample from seven growth stages) of *A. thaliana*. rRNA and other linear RNA were removed using the Ribo-Zero^TM^ Magnetic Kit (Epicentre, Madison, WI, United States) and Ribonuclease R (Epicentre, Madison, WI, United States), respectively. The treated RNA samples were deep-sequenced, resulting in a total of 181.97 Gb of data (151.37 Gb from 13 samples and 30.6 Gb from a mixed sample; average sequencing depth reached approximately 106×), and yielding 795.62 million raw reads that uniquely mapped to the reference genome (*Arabidopsis_thaliana* TAIR10.30 ENSEMBL). After filtering out the contaminated and low-quality reads, 763.81 million clean reads (96.00% of the total raw reads) mapped to the reference genome, indicating the relatively high quality of the sequenced samples (Supplementary Table S2).

### Distribution of CircRNAs in the *A. thaliana* Genome

**FIGURE 1:**
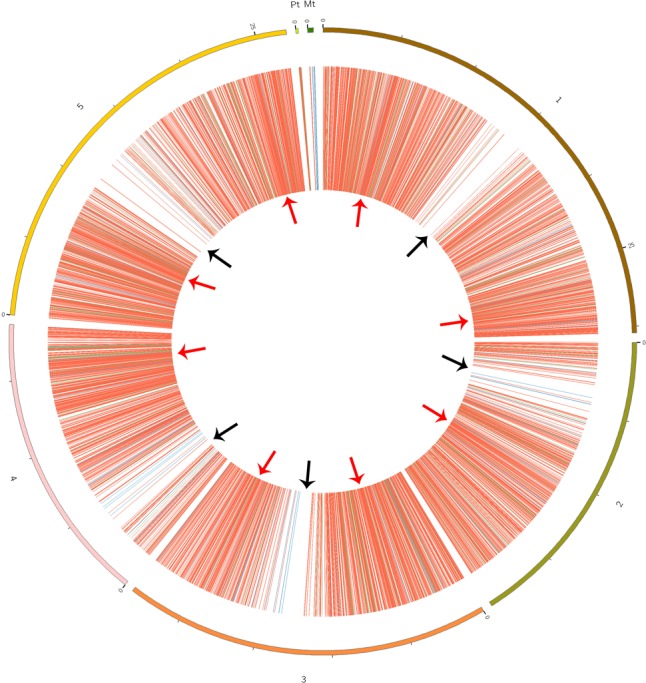
Circos plots showing the identified circRNA distribution in the *Arabidopsis thaliana* genome. Red, green, and blue lines represent exonic, intronic, and intergenic circRNAs, respectively. Red arrows show the genome region with a high density of circRNAs; black arrows show the regions with rare circRNAs.

CircRNAs are recognizable based on back-spliced reads in rRNA-depleted RNA sequencing (RibominusSeq) data. RibominusSeq reads were first mapped to the reference and the circRNAs were then predicted using the find_circ program. In total, 5,861 circRNAs were identified from 14 samples of *A. thaliana*, using the strict threshold of at least two unique back-spliced supporting reads, and quantified. Upon comparison with all *A. thaliana* circRNAs from the plant circbase, 4,586 (78.25%) (Supplementary Table S3) of our identified circRNAs were found. Accordingly, the other 1,275 (21.75 %) circRNAs were regarded as candidate novel circRNAs (Supplementary Table S4). The identified circRNAs were generated from all of the chromosomes, as well as from the mitochondrial and chloroplast genomes (**Figure 1**). However, the circos plot revealed a non-random distribution of circRNAs in the chromosomes. Some chromosomal regions lacked circRNAs (**Figure 1**, black arrows), and some regions had a high density of circRNAs (**Figure 1**, red arrows). The regions with black arrows were around the centromere (the pericentromeric region), where there were low levels of gene density and gene expression (The Arabidopsis Genome Initiative, 2000). A total of 5,861 circRNAs and 54,873 back-spliced reads were identified. In addition, the numbers of circRNAs and back-spliced reads differed among the chromosomes (**Table 2**). There were only 93 circRNAs and 2,560 back-spliced reads on mitochondrial DNA, accounting for about 1.6 and 4.7% of the total numbers of circRNAs and back-spliced reads, respectively. On chromosome 1 and chromosome 5, 1,260 circRNAs (21.5%) and 1,152 circRNAs (19.6%) were found, respectively. The circRNA numbers on chromosomes 2, 3, and 4, and chloroplast DNA ranged from 702 to 936. In addition, compared with other chromosomes, chromosome 5 had more back-spliced reads (15,420, 28.1%). This indicated that circRNAs are the most abundant on chromosomes 1 and 5, less abundant on chromosomes 2, 3, and 4 and chloroplast DNA, and sparsely distributed on mitochondrial DNA.

**Table 2:** Distribution of circRNAs and their reads on different chromosomes in *A. thaliana*.

Chromosome	CircRNAs	Percentage (%)	Reads	Percentage (%)
1	1,260	21.5	10,578	19.3
2	809	13.8	7,078	12.9
3	936	16.0	7,386	13.4
4	702	12.0	5,425	9.9
5	1,152	19.6	15,420	28.1
Mt	93	1.6	2,560	4.7
Pt	909	15.5	6,426	11.7
Total	5,861	100.0	54,873	100.0

*Mt, mitochondria; Pt, chloroplasts*.

### Classification and Conservation of CircRNAs in *A. thaliana*

Among the identified circRNAs, 4,990 (85.1%) were generated from exons of a protein-coding gene, that is, they were exonic circRNAs, meaning that both back-splice sites coincided with known exonic boundaries. A total of 650 circRNAs (11.1%) were derived from intergenic regions. Furthermore, only 221 (3.8%) of the circRNAs were generated by introns (Supplementary Table S5). These results indicated that circRNAs in *A. thaliana* were generated from different genomic regions (**Figure 1**) and mainly from coding regions. Additionally, we also found that most parent genes (2,546) could produce more than one circRNA, while other parent genes (770) produced only one circRNA (Supplementary Table S6).

We further performed reciprocal BLAST analysis to evaluate the conservation of circRNAs, and found that 2,364 and 494 identified circRNAs of *A. thaliana* had sequence similarity with 1,231 and 188 identified circRNAs from *O. sativa* and *S. lycopersicum*, respectively (BlastN, word_size 5, *E* < 1e^-5^). Moreover, 23 and 51 circRNAs also shared sequence similarity with 8 and 37 identified circRNAs from *H. vulgare* and *Z. mays*. In addition, there were 404 circRNAs possessing similar sequences in *A. thaliana*, *O. sativa*, and *S. lycopersicum* (Supplementary Table S7). Interestingly, six circRNAs (Ath_circ_FC1780, Ath_circ_FC3573, Ath_circ_FC3780, Ath_circ_FC1779, Ath_circ_FC1980, and Ath_circ_FC3745) were found in three other species (*O. sativa*, *S. lycopersicum*, and *Z. mays*). These results suggested that the sequences of some circRNAs are conserved among different plant species.

### Functional Annotation Analysis of Parent Genes of CircRNAs in *A. thaliana*

**FIGURE 2:**
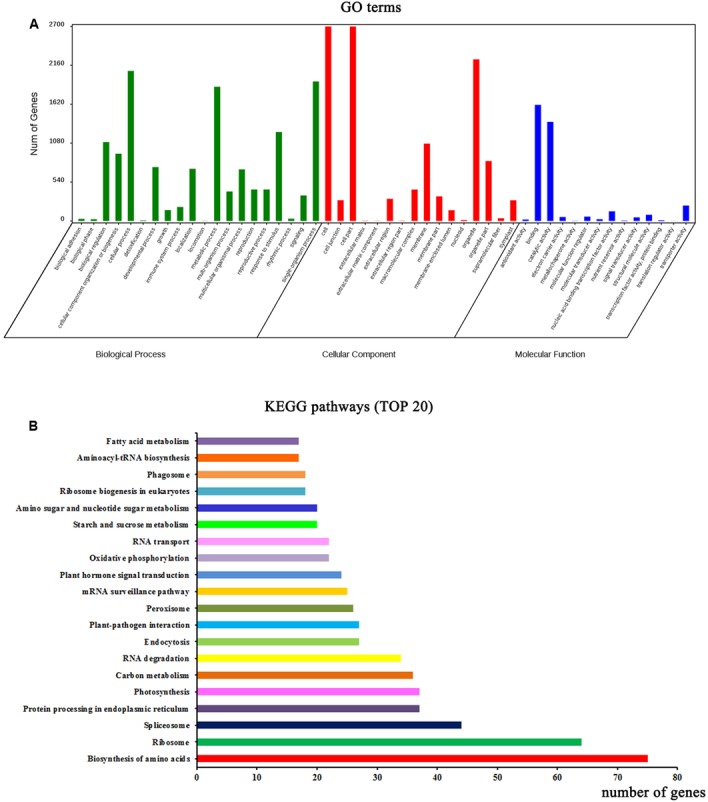
Functional annotations of parent genes (protein-based genes) of circRNAs. **(A)** Gene ontology (GO) classifications of parent genes of circRNAs. **(B)** The top 20 enriched KEGG pathways of circRNA parent genes.

Functional annotation analysis was performed to evaluate the potential functions of the parent genes of circRNAs. GO categories were assigned to the parent genes of circRNAs, and the genes were classified into three GO categories: cellular component, biological process, and molecular function (**Figure 2A**). In the biological process category, some important GO subcategories such as “developmental process,” “reproduction,” and “response to stimulus” were annotated. In the molecular function category, the “antioxidant activity,” “electron carrier activity,” and “signal transducer activity” subcategories were also annotated. The biological interpretations of the circRNA parent genes were further analyzed using the Kyoto Encyclopedia of Genes and Genomes (KEGG) pathway database. As shown in **Figure 2B**, the most highly represented pathways included biosynthesis of amino acids, ribosome, spliceosome, protein processing in endoplasmic reticulum, photosynthesis, and carbon metabolism, implying that many parent genes of circRNAs in *A. thaliana* are involved in protein synthesis and processing, photosynthesis, and carbon metabolism.

### Prediction of miRNA Target Sites in CircRNAs

**FIGURE 3:**
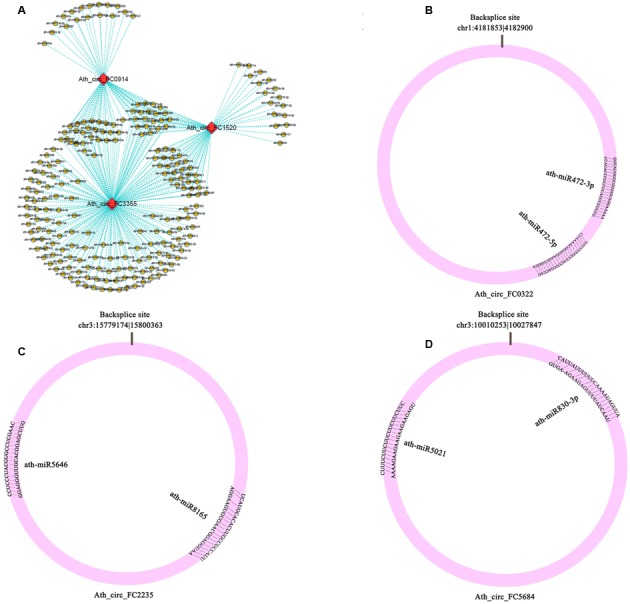
Potential interaction networks of miRNAs and circRNAs. **(A)** Relationship between circRNAs (Ath_circ_FC0914, Ath_circ_FC1520, and Ath_circ_FC3355) and their binding miRNAs. **(B–D)** Schema diagrams show the pairing of each circRNA sequence and the sequence of its targets (Ath_circ_FC0322: ath-miR472-3p, ath-miR472-5p; Ath_circ_FC2235: ath-miR8165, ath-miR5646; and Ath_circ_FC5684: ath-miR830-3p, ath-miR5021).

To determine whether circRNAs in *A. thaliana* could affect post-transcriptional regulation by binding to miRNAs and preventing them from regulating their target mRNAs, we identified miRNA target sites in circRNAs in *A. thaliana*, and found that 39 of 5,861 (0.67%) circRNAs had putative miRNA-binding sites (Supplementary Table S8). Of these 39 circRNAs, 9 had more than one different miRNA-binding site, and the greatest number of miRNA-binding sites (188) was found in Ath_circ_FC3355 (**Figure 3A**). Three circRNAs (Ath_circ_FC0322, Ath_circ_FC2235, and Ath_circ_FC5684) with the miRNA target sites and their pairing miRNA sequences are shown in **Figures 3B–D**. Most of the sequences of circRNAs and their miRNA target sites completely matched. In addition, we analyzed the numbers of circRNA with potential target sites for each miRNA. The results showed ath-miR5021 binding to 14 circRNAs, ath-miR414 binding to 13 circRNAs, and ath-miR5658 binding to 9 circRNAs (Supplementary Table S8). This indicated that some circRNAs in *A. thaliana* have many potential miRNA binding sites, and may be able to affect the post-transcriptional regulation of various genes.

### Validation of CircRNAs in *A. thaliana*

**FIGURE 4:**
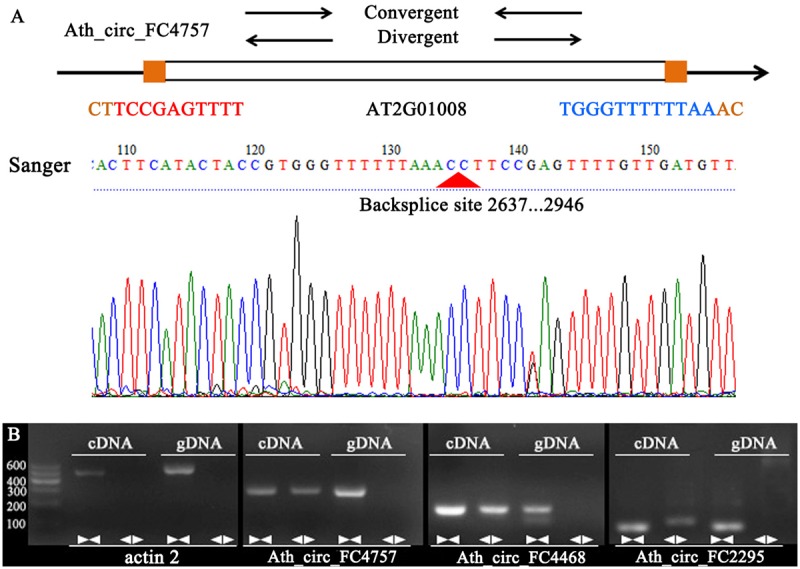
Various experimental strategies validated the circRNAs in *A. thaliana*. **(A)** A representative example of *A. thaliana* circRNAs (Ath_circ_FC4757) showing the validation strategy. Divergent and convergent primers were designed to detect circRNAs, while divergent primers were designed using the “out-facing” strategy. Sanger sequencing further confirmed head-to-tail back-splicing. **(B)** Divergent primers successfully amplified Ath_circ_FC4757, Ath_circ_FC4468, and Ath_circ_FC2295 in cDNA, but failed to do so in genomic DNA. Convergent primers worked on both cDNA and genomic DNA. actin 2: linear control.

To confirm our identification of circRNAs, five randomly selected circRNAs were used in experimental validation using reverse transcription PCR and Sanger sequencing. A set of divergent primers (Supplementary Table S1) was designed for each circRNA and used to amplify both cDNA and genomic DNA. It was expected that positive and negative results of amplification would be obtained for cDNA and genomic DNA, respectively (**Figure 4A**). As a control, convergent primers that should amplify the linear mRNAs were also designed for each circRNA used in verification. The amplified PCR products using divergent primers were sequenced to confirm the presence of the back-spliced junctions. As a result, three circRNAs were validated (Ath_circ_FC4757, Ath_circ_FC4468, and Ath_circ_FC2295) (**Figure 4B**).

**FIGURE 5:**
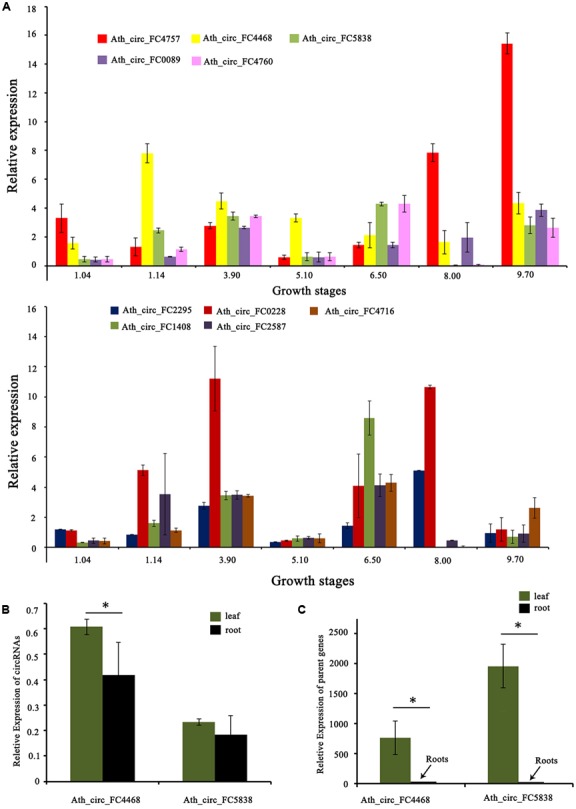
Expression of circRNAs and their parent genes at different developmental stages or in various organs. **(A)** qRT-PCR shows the different expression levels of 10 circRNAs at seven growth stages. **(B)** qRT-PCR shows the different expression levels of Ath_circ_FC4468 and Ath_circ_FC5838 in leaf and root, respectively. **(C)** The different expression levels of the corresponding parent genes of Ath_circ_FC4468 and Ath_circ_FC5838 in leaf and root, respectively. The *y*-axes show relative expression levels analyzed by qRT-PCR. Columns and error bars indicate means and standard deviations of relative expression levels (*n* = 3), respectively. Significant difference (*p* < 0.05) between leaf and root was also evaluated. ^∗^ represents *p* < 0.05.

In addition, we tested the expression levels of 10 selected circRNAs at different growth stages using qRT-PCR. The results showed that these circRNAs at different growth stages had diverse expression patterns. For example, the expression level of Ath_circ_FC1408 increased from growth stages 1.04 to 3.90 and then decreased at growth stage 5.10. In addition, the peak value occurred at growth stage 6.50 whereas the lowest value was at growth stage 8.00 (**Figure 5A**). Moreover, we selected two circRNAs (Ath_circ_FC4468 and Ath_circ_FC5838) with parent genes related to photosynthesis to test their expression levels in leaves and roots. As shown in **Figures 5B,C**, both of the two circRNAs, as well as their parent genes, were expressed more highly in leaves than in roots.

## Discussion

The expression of some circRNAs is often cell-type, tissue-, and developmental-stage-specific, suggesting that circRNAs are important functional molecules (Dang et al., 2016; Gao et al., 2016). Thousands of circRNAs have been identified in mammals, fish, worms, insects, fungi, protists, and human (Danan et al., 2012; Salzman et al., 2012; Memczak et al., 2013; Zhang et al., 2013; Wang et al., 2014; Dang et al., 2016), and this number is rapidly increasing. In plants, circRNAs in *A. thaliana*, *O. sativa*, tomato, and wheat were predicted from public RNA sequencing data or RNA extracted from a plant at a particular stage of development (Lu et al., 2015; Ye et al., 2015; Zuo et al., 2016; Wang et al., 2017). Here, we report genome-wide identification and analysis of circRNAs from 14 ssRNA-seq libraries for RNA samples from four different growth stages, five organs, and four stress treatments, as well as a mixed RNA sample in *A. thaliana*. We obtained 5,861 circRNAs including 1,275 novel ones. Our data demonstrate the presence of circRNAs over the whole of the genome of *A. thaliana* and provide important complementary plant circRNAs for further classification, analysis of their properties, and functional research. In addition, sequence conservation analysis among four species suggested that the conservation of circRNAs in plants is probably widespread.

CircRNAs are primarily derived from the exons of protein-coding genes, although they can also arise from intronic, intergenic, and untranslated regions, ncRNA loci, and from locations antisense to known transcripts (Jeck et al., 2013; Memczak et al., 2013; Ye et al., 2015). In our study, the predicted circRNAs were predominantly exonic circRNAs. As the exonic circRNAs and their parent genes exhibit significant positive correlations in their expression levels, this could be a result of *cis*-transcriptional promotion of circRNAs on their parent genes (Li et al., 2015). This suggests that the non-random distribution of circRNAs on different chromosomes is associated with gene activity, especially in the pericentromeric region. In addition, exonic circRNAs may be subject to splicing in order to remove the intervening introns either before or after circularization. However, new research has demonstrated that EIciRNAs are specified by the presence of a retained intron, and can interact with U1 small nuclear ribonucleoproteins and enhance the expression of their parent genes (Li et al., 2015). Studies in rice have shown that multiple circRNAs can originate from a single gene (Lu et al., 2015). In this study, there were also multiple circRNAs from one parent gene, indicating that alternative back-splicing also occurs in *A. thaliana*. In addition, the emergence of different types of circRNA is also associated with alternative splicing (Zhang et al., 2014; Gao et al., 2016). As the alternative splicing events in circRNAs are not consistent with the corresponding mRNAs (Gao et al., 2016); therefore, the biogenesis, regulation, and function of these alternatively spliced circRNAs in plants are worthy of further study.

Many studies have reported that some circRNAs are conserved across species. For example, Jeck et al. (2013) identified 69 murine circRNAs in precisely orthologous locations to human cirRNAs. Similarly, recent study reported almost 600 circRNAs in which genomic location in human were overlapped with the syntenic region in mouse (Legnini et al., 2017). In addition, the exonic sequences known to circularize seem to be more conserved at the third codon position (Ebbesen et al., 2016). These results indicated that some circRNAs in mammal are stable and conserved. In plants, Ye et al. (2015) firstly explored the conservation of circRNAs in two model plants, *O. sativa* and *A. thaliana*, and identified more than 300 orthologous parent genes generating circRNAs from a similar position, implying that plant circRNAs have the conservation feature as in animals. More recently, a study on soybean circRNAs found that 551 parent gene pairs producing exonic circRNAs among *O. sativa*, *A. thaliana*, and soybean were orthologs (Zhao et al., 2017). In our study, we found 404 identified circRNAs derived from 190 parent genes in *A. thaliana* have sequence similarity with those in *O. sativa* and *S. lycopersicum*. Also, six identified circRNAs in *A. thaliana*, *O. sativa*, *S. lycopersicum*, and *Z. mays* showed sequence similarity. These results suggested the sequence conservation of circRNAs may exist widely across plant species. However, the orthologous parent genes producing circRNAs from orthologous splice donor and splice acceptor sites of identical exons in a broader plant species, and particularly the potential function of these conserved circRNAs need further study in the plant kingdom.

Previous research has indicated that circRNAs can regulate transcription and circRNA production may occur post-transcriptionally (Liang and Wilusz, 2014). Chao et al. (1998) first proposed that the formation of circRNAs traps the transcripts arising from the linear gene in a nonfunctional form and prevents the existence of certain normal linear transcripts that could be translated. Thus, circRNAs can act as an “mRNA trap” by sequestering the translation start site, leaving a non-coding linear transcript and thereby reducing the expression level of the parent genes (Jeck and Sharpless, 2014). These results suggest a potential role for plant circRNAs in the regulation of their parent genes. To understand further the regulation of circRNA transcription in *A. thaliana*, GO and KEGG analyses were performed to annotate the biological functions of the parent genes of circRNAs. We noted that some important functions related to developmental processes, reproduction, responses to stimuli, protein synthesis, photosynthesis, and carbon metabolism were annotated in the GO and KEGG databases. These results suggest that these circRNAs generated from these parent genes might be also involved in many fundamental metabolic processes in plant growth, development, and reproduction. This is reasonable as gene expression is readily affected by developmental processes and the environment, and plant circRNAs display diverse expression patterns (Ye et al., 2015). According to previous reports (Lu et al., 2015; Ye et al., 2015; Wang et al., 2017) and the present study (**Table 2**), although a large number of back-splicing sites could be identified, far fewer circRNAs were obtained. In addition, the expression of circRNAs at different developmental stages or different organs displayed rapid changes and variations, and the co-expression of circRNAs at different stages and in different tissues is very limited (Supplementary Table S9). Therefore, it is difficult to determine the developmental stage- and tissue-specific circRNAs, even if biological replicates are used.

There is a regulatory mechanism called endogenous target mimicry (eTM), which involves binding to the corresponding miRNAs to block the binding of a specific transcript, leading to an increase in mRNA expression (Karakülah et al., 2016). Previous studies in plants have reported that lncRNAs could bind to miRNAs as target mimics (Franco-Zorrilla et al., 2007). Although circRNAs are not linear like lncRNAs, a small proportion of plant circRNAs was potential target mimics of miRNAs because the genomic regions that produce circRNAs contain miRNA binding sites (Ye et al., 2015; Wang et al., 2017). This mechanism is similar to miRNA sponges in animals in that circRNAs can act as decoys for miRNAs to regulate gene expression at the epigenetic level (Hansen et al., 2013; Memczak et al., 2013). In mouse, *Sry* circRNA harboring 16 putative binding sites acted as an inhibitor of miR138, and ciRS-7 could suppress miR-7 activity, suggesting circRNAs as miRNA sponges (Hansen et al., 2013). This sponge effect was also found in *Drosophila* that highly conserved miRNA binding sites could overlap with circRNA production (Westholm et al., 2014). Although in plants many miRNA binding sites within circRNAs have been predicted, no direct evidence has proved that circRNAs act as miRNA sponges (Lu et al., 2015; Ye et al., 2015; Wang et al., 2017). For example, in soybeans, 2,134 circRNAs contained predicted binding sites for 92 miRNAs (Zhao et al., 2017), and 1,861 circRNAs from PlantcircBase were predicted as putative miRNA sponges (Chu et al., 2017). In this study, we found that 39 circRNAs had putative miRNA-binding sites, and the number of miRNA-binding sites among circRNAs varies considerably, suggesting that some circRNAs are potential miRNA targets in *A. thaliana*. This indicated that plant circRNAs might play roles in gene expression regulation by interacting with miRNAs. For example, miR172 plays a crucial role in regulating the transitions between developmental stages and in specifying floral organ identity, as well as in regulating legume–rhizobia nitrogen-fixing symbiosis (Zhu et al., 2009; Zhu and Helliwell, 2011; Íñiguez et al., 2015). Furthermore, a circRNA has been predicted to be a target mimic of miR172 in rice and tomato (Lu et al., 2015; Zuo et al., 2016). In our results, three circRNAs were also found to have miR172 binding sites, indicating that the circRNAs interacting with miRNAs to regulate gene expression may be conserved in plants.

Most circRNAs were considered to be non-coding RNAs. Recently, Yang et al. (2017) reported that m6A-driven translation of circRNAs is widespread in human cells, with hundreds of endogenous circRNAs having translation potential. Moreover, a circRNA (circ-ZNF609) containing an open-reading frame can be translated into a protein, providing an example of a protein-coding circRNA in murine and human myoblasts (Legnini et al., 2017). It was also reported in fly that a group of circRNAs is associated with translating ribosomes and that a circRNA generated from the muscleblind locus encodes a protein (Pamudurti et al., 2017). Thus, it appears that circRNA translation is widespread in eukaryotes. In our study, we found that most of the identified circRNAs were derived from exons (85.16%), raising the intriguing possibility of circRNA translation to produce proteins. Therefore, the issues of how circRNAs are translated and which functions circRNA-coding proteins have in plants might be promising research areas warranting further investigation.

## Author Contributions

GC and BJ designed the research. GC, JC, ZL, and LW performed the research; GC, YZ, and BJ analyzed the data. GC and BJ wrote the manuscript.

## Conflict of Interest Statement

The authors declare that the research was conducted in the absence of any commercial or financial relationships that could be construed as a potential conflict of interest.

## Footnotes

1 http://ibi.zju.edu.cn/plantcircbase/index.php

2 https://www.blast2go.com/

3 https://github.com/carringtonlab/TargetFinder
