# Supplementary material for: Genome-Wide Identification of Circular RNAs in Arabidopsis thaliana
Source: Front Plant Sci. 2017 Sep 27;8:1678. doi: 10.3389/fpls.2017.01678 (PMC5623955; doi:10.3389/fpls.2017.01678)
Supplement: Supplementary file 1 [file Table_1.DOCX]

**Supplementary Table 1. Primer list of validated circRNAs.**

| No. | Name of circRNA | Divergent primers | Convergent primers |
| --- | --- | --- | --- |
| 1 | Ath_circ_FC4757 | F: GGCGTGCTTCGTTGGAA  R: CATCCAACCTAGGCGAGAC | F: CCTTCCGAGTTTTGTTGATG  R: GTTTAAAAAACCCACGGTAGTATGA |
| 2 | Ath_circ_FC4468 | F: CATAAGGACCGCCGTTG R: CTTGGTGTAGCTTGTTATATGGG | F: CGCATACCCAGACGGAAACT  R: ATGGCTATACAACGGCGGTC |
| 3 | Ath_circ_FC2295 | F: AAGCAGTGACAGATCCGTATG R: GTCCAACTGAAATGTATCCACC | F: CACCTCATGTCCTAGCTCAAGTT  R: CGGATCTGTCACTGCTTCC |
| 4 | Ath_circ_FC1408 | F: CTAGCTGACCGGTTTGGAGTT  R: CTTGTGAGCAGGGACATCCA |  |
| 5 | Ath_circ_FC2587 | F: AGATCCCAGAGTACTTAAACGGT R: ATCCAAAAGACCATCAGGCAAG |  |
| 6 | Ath_circ_FC0228 | F: CCATTTGTGTATAAACATCTCGG  R:CTTTGCGCTATTGGCCC |  |
| 7 | Ath_circ_FC0089 | F: AGGGAAGTTGTGAGCATTACG  R: CGAGCAATATAAGCCTTCTTGG |  |
| 8 | Ath_circ_FC4760 | F: AGTGGCGTCGAGCTGTGAT  R:CAATAAGTTGGTGGAACACTGGCT |  |
| 9 | Ath_circ_FC4716 | F: GATCCGTCCACTGCTGAGAG  R: ACGTCGAGCTCAGTAACGTG |  |
| 10 | Ath_circ_FC5838 | F: CATACCAAGGTTAGCACGGTTA  R: TGCTGTTGAGGCTCCATCTA |  |
| 11 | Actin2 | F: TCCTTCTGGTTCATCCCAAC  R：GCAGAGCGGGAAATTGTAAG |  |
