# Supplementary material for: Genome-Wide Identification of Circular RNAs in Arabidopsis thaliana
Source: Front Plant Sci. 2017 Sep 27;8:1678. doi: 10.3389/fpls.2017.01678 (PMC5623955; doi:10.3389/fpls.2017.01678)
Supplement: Supplementary file 5 [file Table_5.DOCX]

| Type | 1 | | 2 | 3 | 4 | 5 | Mt | Pt | Sum |
| --- | --- | --- | --- | --- | --- | --- | --- | --- | --- |
| Exonic | | 1143 | 724 | 855 | 644 | 1052 | 36 | 536 | 4990 |
| Intronic | | 53 | 42 | 36 | 27 | 49 | 1 | 13 | 221 |
| Intergenic | | 64 | 43 | 45 | 31 | 51 | 56 | 360 | 650 |
| Sum | | 1260 | 809 | 936 | 702 | 1152 | 93 | 909 | 5861 |

**Table S 5** Types of circRNAs in different chromosomes, mitochondria and chloroplasts of *A. thaliana*

Mt: mitochondria, Pt: chloroplasts
